# Ascl2 activation by YAP1/KLF5 ensures the self-renewability of colon cancer progenitor cells

**DOI:** 10.18632/oncotarget.22673

**Published:** 2017-11-27

**Authors:** Xiaolong Wei, Jun Ye, Yangyang Shang, Haoyuan Chen, Shanxi Liu, Li Liu, Rongquan Wang

**Affiliations:** ^1^ Institute of Gastroenterology of PLA, Southwest Hospital, Third Military Medical University, Chongqing, China

**Keywords:** achaete scute-like 2, Wnt signaling, Hippo signaling, colorectal cancer, progenitor cells

## Abstract

Achaete scute-like 2 (Ascl2) is the Wnt signaling target, its regulation by other signaling is undefined. Now we demonstrated that CD133^+^/CD44^+^ cell population from HT-29 or Caco-2 cells exhibited cancer stem cell (CSC) properties with highly expressed Ascl2, which is related to the Hippo signaling pathway. YAP1 interference in CD133^+^/CD44^+^ HT-29 or Caco-2 cells reduced their proliferation, colony-forming ability and tumorsphere formation *in vitro* and inhibited the ‘stemness’-associated genes and Ascl2 expression. Enforcing YAP1 expression in HT-29 or Caco-2 cells triggered the opposite changes. Ascl2 interference reversed the phenotype of YAP1-enforced expressed HT-29 or Caco-2 cells. Krüppel-like factor 5 (KLF5) protein, not KLF5 mRNA levels, were increased due to YAP1 overexpression which is reported to prevent KLF5 degradation. Co-immunoprecipitation (Co-IP) assays demonstrated that YAP1 bound with KLF5 in HT-29 and Caco-2 cells. Luciferase and chromatin immunoprecipitation (ChIP) assays indicated that both YAP1 and KLF5 bound to the first two loci with GC-boxes in Ascl2 promoter and induced Ascl2 transcription. The decreased Ascl2 transcription by YAP1 interference required an intact KLF5 binding site (GC-box) within Ascl2 promoter, KLF5 knockdown reduced YAP1 binding and Ascl2 luciferase reporter activity upon YAP1 overexpression. Positive correlation among YAP1 and Ascl2 mRNA levels was observed in colorectal cancer (CRC) samples. Thus, our study demonstrated that Ascl2, a fate decider of CRC progenitor cells can be activated by the Hippo signaling pathway in CRC progenitor cells, and ensured their self-renewability.

## INTRODUCTION

Colorectal cancer (CRC) is a leading cause of morbidity and mortality in developed countries [1]. Cancer stem cells (CSCs), which exhibit stem-like features, sustain tumor formation, cause metastasis, and are resistant to therapy, have been proposed to explain the functional heterogeneity and carcinogenesis [2-5]. Protein-coding genes and their products participate in the ‘stemness’ maintenance and tumorigenicity of CRC progenitor cells [6-8]. Thus, it is pivotal to identify the signaling pathways involved in CRC progenitor cells to develop novel reagents that target the refractory CRC progenitor cell population [9].

Achaete scute-like 2 (Ascl2/Mash2/Hash2), a helix-loop-helix transcription factor and downstream target of Wnt signaling, plays a critical role in intestinal Lgr5^+^ cryptic stem cells and CRC progenitor cells [10, 11]. Ascl2 is abundantly expressed in colorectal cancer; has the potential to shift the hierarchy of stem and progenitor cells during liver metastasis, resulting in self-renewal rather than differentiation; and potentially affects the clinical behavior of these tumors [12-14]. Ascl2 can also initiate T-helper cell development, trophoblast progenitor differentiation of mouse trophoblast stem cells, and differentiation of intestinal neoplastic epithelial cells that results in a goblet cell phenotype [15-17]. Mash2 in trophoblast stem cells can be transcriptionally increased by Tssc3, HIF-1α, HIF-2α and OVO-like 1 (OVOL1), and Mash2 expression is decreased due to a Mst1/2 double knockout during mouse placental development [16, 18-20]. In CRC, Ascl2 is significantly induced by either the chemokine receptor CXCR4 or KIAA1199, hypoxia-induced HIF-1α [21-23]. However, the molecular mechanism of Ascl2 overexpression in CRC progenitor cells is still unclear.

The mammalian Hippo signaling pathway, short for MST1/2-LATS1/2-YAP1/TAZ-TEAD1-4, is a critical pathway involved in determining cell growth rate and organ size [24-26]. MST1 and MST2 (MST1/2) phosphorylate LATS1/2 and Mob1, leading to their activation; LATS1/2 phosphorylates YAP1/TAZ, which causes a cytoplasmic accumulation of the phosphorylated YAP1/TAZ and sequesters its oncogenic function [27-29]. The unphosphorylated YAP1/TAZ translocates to the nucleus and binds with TEAD1-4 (TEA domain DNA-binding transcription factors 1-4) and other transcription factors, inducing transcriptional activity that causes cell proliferation and differentiation. YAP1/TAZ activation is widespread in many human tumors and has been shown to be essential for cancer initiation, progression, and metastasis, they function as transcriptional co-activators that shuttle between the cytoplasm and the nucleus, where they induce expression of cell-proliferative and anti-apoptotic genes via interactions with transcription factors [30-32].

In 2010, crosstalk study between Wnt and Hippo signaling has been suggested, and evidence of cytoplasmic TAZ inhibiting canonical Wnt signaling has been provided, highlighting that the Hippo pathway can control other signaling cascades [33]. The β-catenin-YAP1-TBX5 (transcription factor) complex is essential to the transformation and survival of β-catenin-driven cancers [34]. These two pathways are known to be important for epithelial development and homeostasis, and there is accumulating evidence that the Hippo cascade engages in crosstalk with Wnt signaling in epithelial tissues [35-37]. Essential crosstalk between Hippo and Wnt occurs during epithelial transcriptional control. YAP1 reprograms Lgr5^+^ cryptic stem cells by inhibiting the Wnt homeostatic program while inducing a regenerative program that includes the activation of EGFR signaling, which drives cancer initiation [38]. However, understanding the molecular mechanisms connecting these signals will require further investigation.

Here, we provided a study that YAP1 combined with KLF5, further bound to the Ascl2 promoter, and then transcriptionally activated Ascl2 expression. This mechanism had an impact on the self-renewability of colon cancer progenitor cells. A correlation between YAP1 and Ascl2 was present not only in the CD133^+^CD44^+^ CRC cell population but also in colon cancer tissue samples. Ascl2 regulation by YAP1 provided evidence that this Wnt target gene can also be transcriptionally activated by YAP1, an effector of Hippo signaling in CRC progenitor cells, and YAP1-activated Ascl2 expression in CRC progenitor cells controlled their cell state stability and conferred their ability to self-renewability.

## RESULTS

### CD133^+^CD44^+^ HT-29 or Caco-2 cells had higher abilities to proliferate and form colonies and tumorspheres

**Figure 1:**
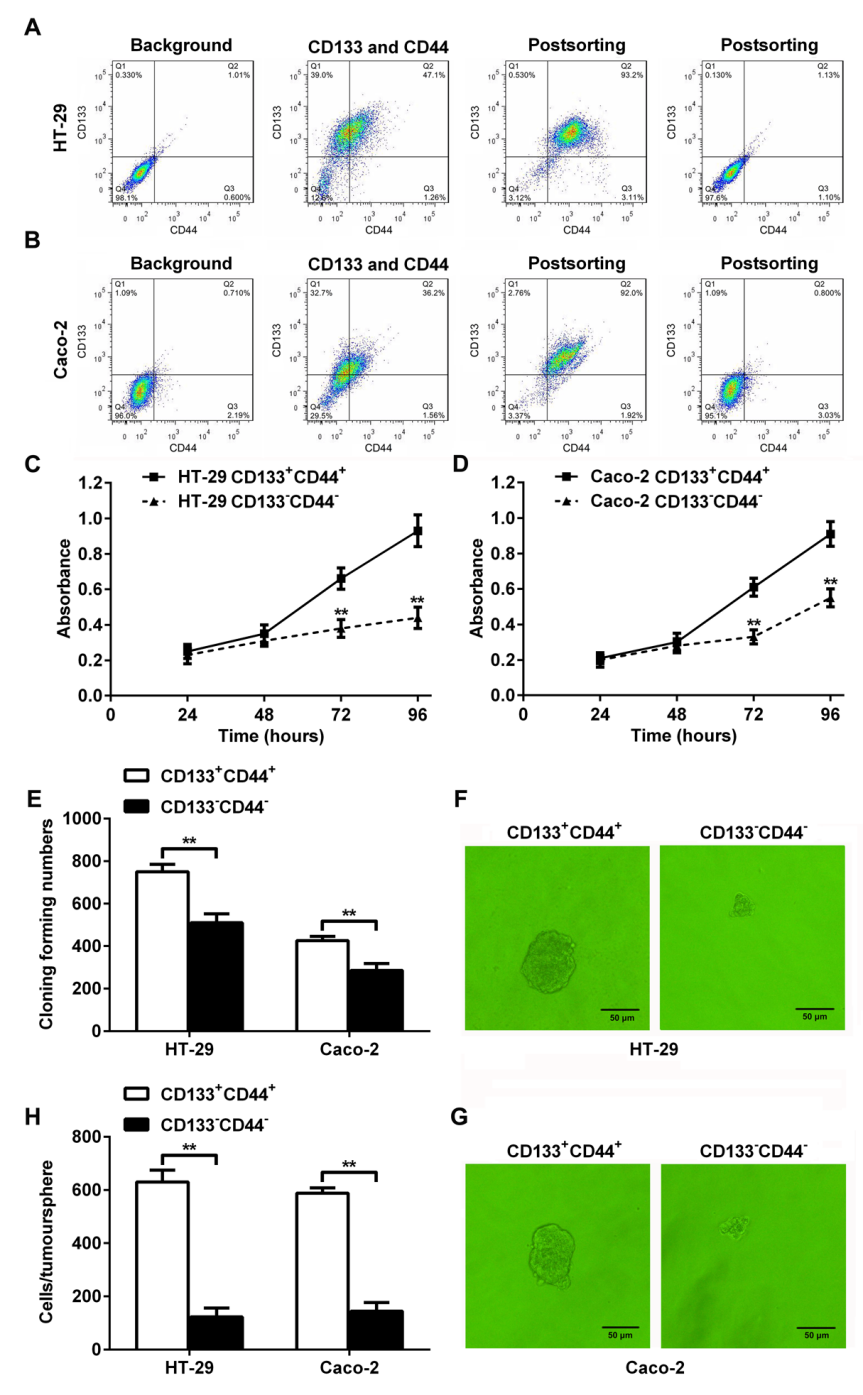
Characterization of CD133^+^CD44^+^ and CD133^-^CD44^-^ CRC cells (**A and B**) Fluorescence-activated cell sorting and flow cytometry. CRC cells HT-29 (A) and Caco-2 (B) were separated into CD133^+^CD44^+^ and CD133^-^CD44^-^ CRC cell population. (**C and D**) The proliferation rates of CD133^+^CD44^+^ and CD133^-^CD44^-^ HT-29 (C) or Caco-2 (D) cells from days 1 through 4 after seeding (^**^: *P<*0.01). **(E)** The colony formation assay indicated that more colonies were formed in the CD133^+^CD44^+^ CRC cell population than in the CD133^-^CD44^-^ CRC cell population (^**^: *P<*0.01). (**F and G**) Tumorsphere formation of CD133^+^CD44^+^ or CD133^-^CD44^-^ CRC cell population from HT-29 (F) or Caco-2 (G) cells. **(H)** The cell numbers per tumorsphere in CD133^+^CD44^+^ and CD133^-^CD44^-^ HT-29 or Caco-2 cells (^**^: *P<*0.01).

Fluorescence-activated cell sorting and flow cytometry was used to separate HT-29 and Caco-2 cells into CD133^+^CD44^+^ and CD133^-^CD44^-^ CRC cell population (Figure 1A and 1B). The proliferation rates of the CD133^+^CD44^+^ HT-29 or Caco-2 cells from days 3 to 4 after seeding were significantly higher than CD133^-^CD44^-^ HT-29 or Caco-2 cells (Figure 1C and 1D). More colonies were formed in the CD133^+^CD44^+^ HT-29 or Caco-2 cells than CD133^-^CD44^-^ HT-29 or Caco-2 cells, as determined by the colony formation assay (Figure 1E). Larger tumorspheres were formed in the CD133^+^CD44^+^ HT-29 (Figure 1F) or Caco-2 (Figure 1G) cells, and the cell numbers per tumorsphere in the CD133^+^CD44^+^ HT-29 or Caco-2 cells were significantly higher than CD133^-^CD44^-^ HT-29 or Caco-2 cells (Figure 1H). The results indicated that the CD133^+^CD44^+^ CRC cell population represented colon cancer progenitor cells.

### Expression of Ascl2, KLF5, Hippo signaling and ‘stemness’-associated genes in CD133^+^CD44^+^ and CD133^-^CD44^-^ CRC cells

**Figure 2:**
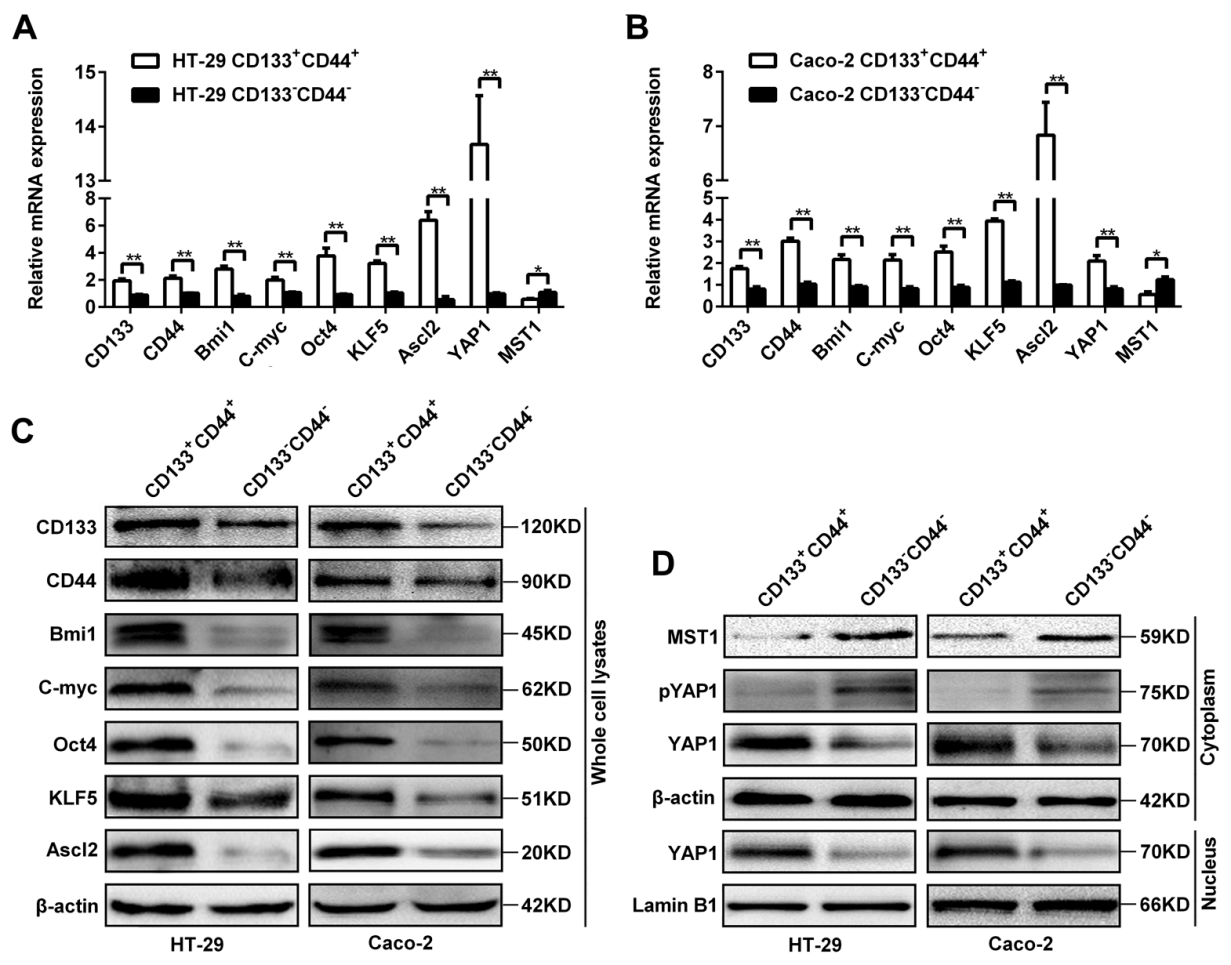
Expression of Ascl2, KLF5, the Hippo signaling pathway, and ‘stemness’-associated genes in CD133^+^CD44^+^ or CD133^-^CD44^-^ CRC cells (**A and B**) Relative mRNA expression levels of Ascl2, KLF5, the Hippo signaling pathway, and ‘stemness’-associated genes in CD133^+^CD44^+^ or CD133^-^CD44^-^ HT-29 (A) and Caco-2 (B) cells (^*^: *P<*0.05, ^**^: *P<*0.01). **(C)** The protein expression levels of Ascl2, KLF5 and ‘stemness’-associated genes in CD133^+^CD44^+^ and CD133^-^CD44^-^ HT-29 or Caco-2 cells; β-actin was used as a loading control. **(D)** The protein expression levels from the Hippo signaling pathway: MST1, phosphorylated YAP1 and YAP1 in the cytoplasm or the nucleus of CD133^+^CD44^+^ and CD133^-^CD44^-^ HT-29 or Caco-2 cells; β-actin was used as a loading control for cytoplasmic proteins, and Lamin B1 was used as a loading control for nucleus proteins.

There were higher mRNA levels of ‘stemness’-associated genes (CD133, CD44, Bmi1, C-myc and Oct4), Ascl2, KLF5 and YAP1, and lower MST1 level in CD133^+^CD44^+^ HT-29 and Caco-2 cells compared with CD133^-^CD44^-^ HT-29 and Caco-2 cells (Figure 2A and 2B). The protein levels of Ascl2, KLF5 and ‘stemness’-associated genes in CD133^+^CD44^+^ HT-29 and Caco-2 cells were higher than CD133^-^CD44^-^ HT-29 and Caco-2 cells (Figure 2C). The YAP1 protein levels in both the cytoplasm and the nucleus of CD133^+^CD44^+^ HT-29 and Caco-2 cells were higher than CD133^-^CD44^-^ HT-29 and Caco-2 cells, whereas MST1 and phosphorylated YAP1 were present predominantly in the cytoplasm of CD133^-^CD44^-^ HT-29 and Caco-2 cells (Figure 2D). The data indicated that an accordant increase of ‘stemness’-associated genes, Ascl2, KLF5, and YAP1 expression in CD133^+^CD44^+^ HT-29 and Caco-2 cells. MST1 and phosphorylated YAP1 were higher in the cytoplasm of CD133^-^CD44^-^ HT-29 and Caco-2 cells than CD133^+^CD44^+^ HT-29 and Caco-2 cells (Figure 2D).

### Proliferation, CD133^+^CD44^+^ percentages, and Ascl2, KLF5 and ‘stemness’-associated genes expressions in YAP1 interfered colon cancer cells

**Figure 3:**
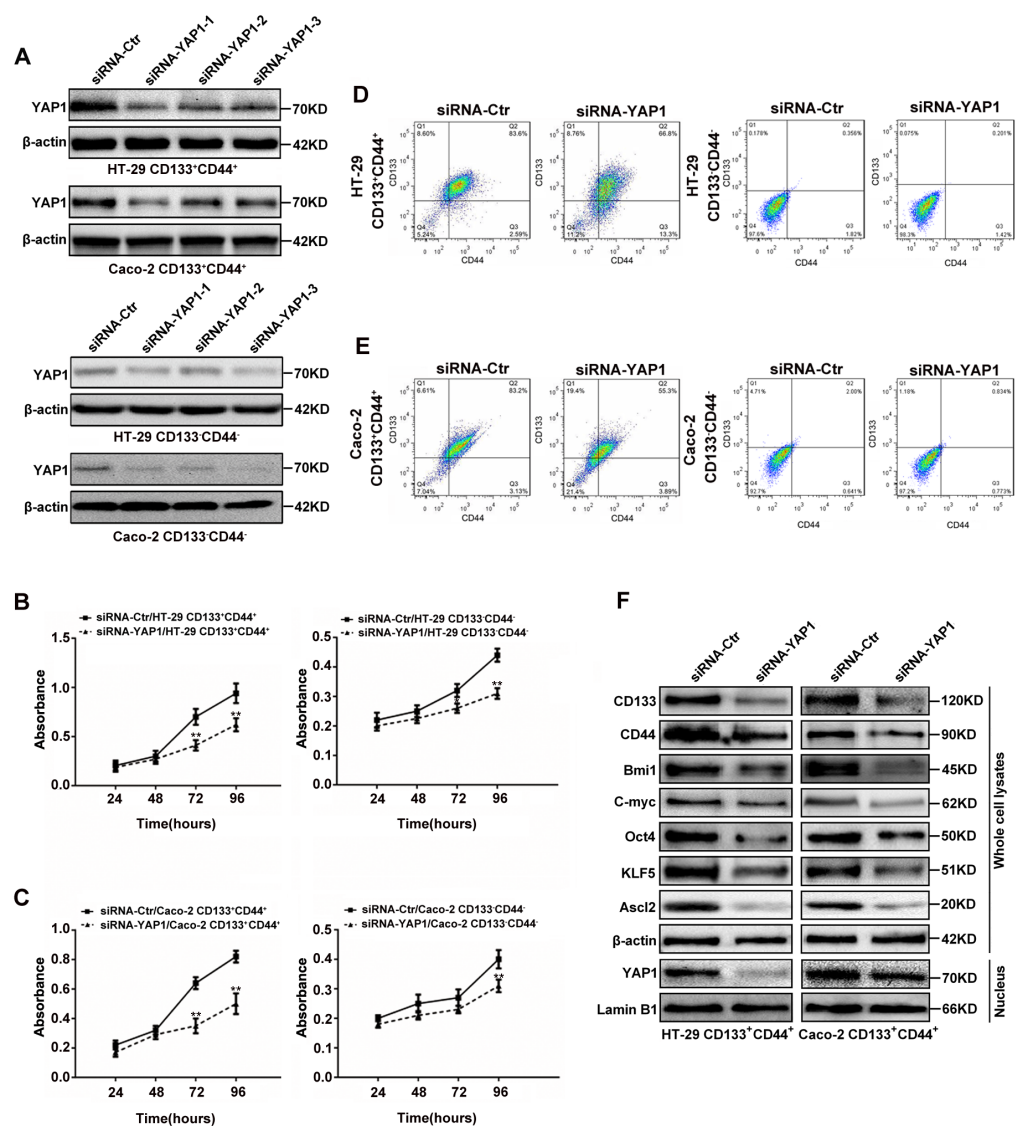
YAP1 knockdown in CD133^+^CD44^+^ or CD133^-^CD44^-^ CRC cells affected their proliferation, CD133^+^CD44^+^ percentages, relative gene expression **(A)** Western blots of YAP1 in CD133^+^CD44^+^ or CD133^-^CD44^-^ CRC cells transiently transfected with YAP1 siRNA1 (siRNA-YAP1-1), siRNA2 (siRNA-YAP1-2) or siRNA3 (siRNA-YAP1-3); β-actin was used as a loading control. (**B and C**) The proliferation rates of CD133^+^CD44^+^ and CD133^-^CD44^-^ HT-29 (B) or Caco-2 (C) cells transfected with siRNA-YAP1-1 from days 1 through 4 after seeding (^**^: *P<*0.01). (**D and E**) The CD133^+^CD44^+^ percentages determined by flow cytometry in YAP1-interfered CD133^+^CD44^+^ or CD133^-^CD44^-^ CRC cells. **(F)** The protein expression levels of Ascl2, KLF5 and ‘stemness’-associated genes in the whole cell lysates of CD133^+^CD44^+^ CRC cells transfected with siRNA-YAP1-1; β-actin was used as a loading control. YAP1 nuclear accumulation was inhibited in siRNA-YAP1-1-transfected CD133^+^CD44^+^ CRC cells; Lamin B1 was used as an internal control for the nuclear fraction.

The results from screening for the most efficient siRNA sequence to target YAP1 were shown in Figure 3A, siRNA-YAP1-1 exerted the most efficient YAP1 interference in both CD133^+^CD44^+^ and CD133^-^CD44^-^ CRC cells, and it was then used in further experiments. The proliferation rates of CD133^+^CD44^+^ and CD133^-^CD44^-^ CRC cells from HT-29 (Figure 3B) or Caco-2 (Figure 3C) cells from days 1 to 4 after transfected with siRNA-YAP1-1 were based on MTT assays. The proliferation rates of YAP1 interfered CD133^+^CD44^+^CRC cells were significantly decreased from days 3 to 4 (*P*<0.01), whereas, the proliferation rates of YAP1 interfered CD133^-^CD44^-^ CRC cells significantly decreased at day 4 (*P*<0.01), and decreased at day 3 only in YAP1 interfered CD133^-^CD44^-^ HT-29 cells (*P*<0.05). Only 66.8% and 55.3% were CD133 and CD44 positive in YAP1 interfered CD133^+^CD44^+^ HT-29 (Figure 3D) and Caco-2 (Figure 3E) cells compared with 83.6% and 83.2% being CD133 and CD44 positive in mock CD133^+^CD44^+^ CRC cells, respectively. The CD133^+^CD44^+^ percentages were unaltered despite YAP1interference in CD133^-^CD44^-^ CRC cells (Figures 3D and 3E). YAP1 knockdown in CD133^+^CD44^+^ CRC cells led to the reduction of KLF5, Ascl2 and ‘stemness’-associated genes expression and inhibited the accumulation of YAP1 in the nucleus (Figure 3F). The protein expression levels of KLF5, Ascl2 and ‘stemness’-associated genes in CD133^-^CD44^-^ HT-29 and Caco-2 cells were significantly lower than CD133^+^CD44^+^ HT-29 and Caco-2 cells (Figure 2C) and not altered despite YAP1 interference using siRNA-YAP1-1 in CD133^-^CD44^-^ HT-29 and Caco-2 cells (data not shown). The data provided evidence that YAP1 is exclusively essential for maintaining the ‘stemness’ of CD133^+^CD44^+^ HT-29 and Caco-2 cells, including their proliferation, CD133^+^CD44^+^ percentages, and ‘stemness’-associated genes expression. However, it was still unknown whether YAP1 knockdown inhibited the ‘stemness’ of CD133^+^CD44^+^ HT-29 and Caco-2 cells via KLF5-dependent Ascl2 inhibition.

### YAP1-enforced expression in HT-29 and Caco-2 cells increased their proliferation, colony formation, the percentages of CD133^+^CD44^+^ population, and tumorsphere formation

**Figure 4:**
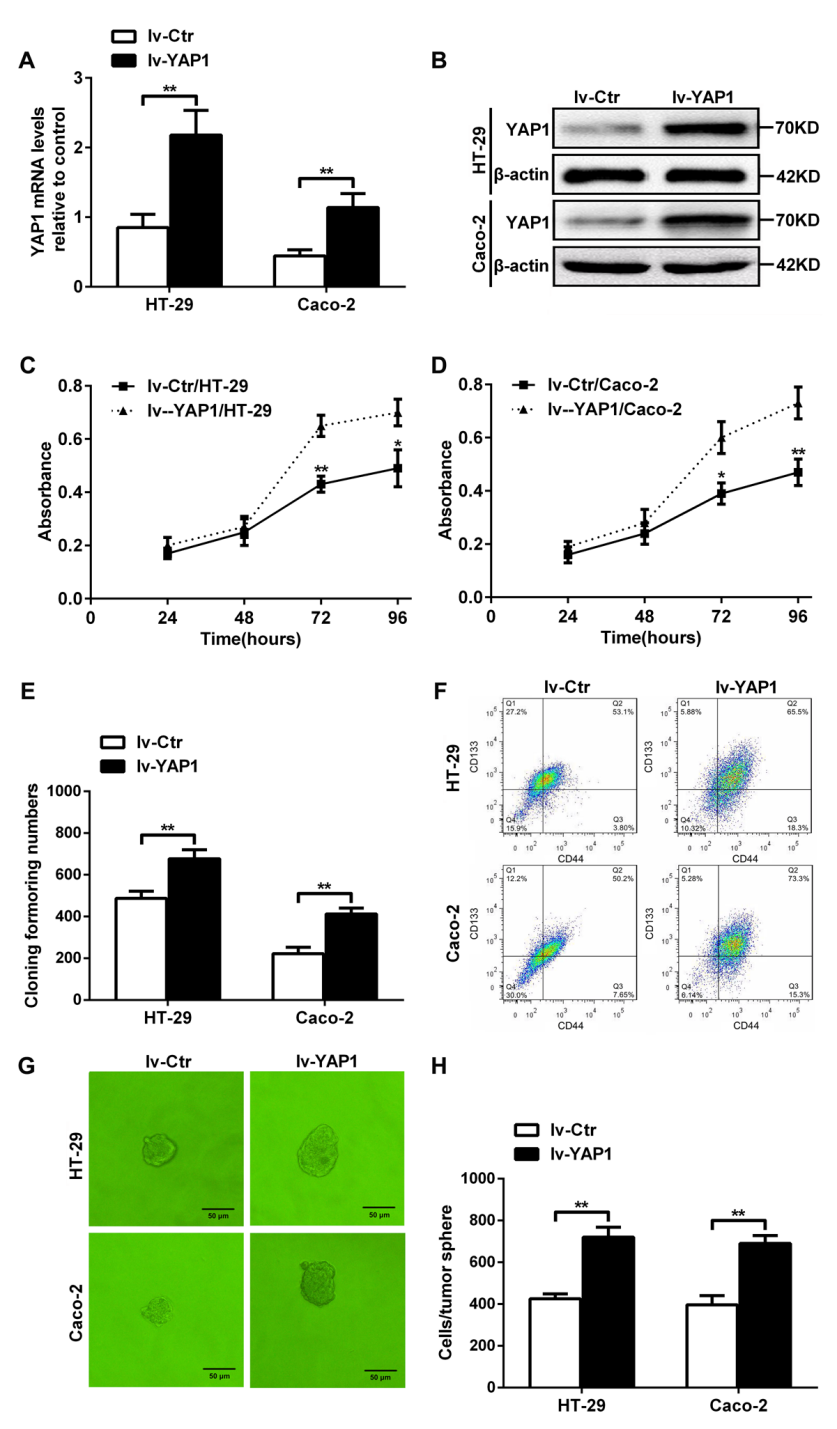
YAP1-enforced expression in HT-29 and Caco-2 cells increased their proliferation, colony formation ability, the CD133^+^CD44^+^ percentages, and tumorsphere formation **(A)** Relative YAP1 mRNA expression levels in lv-YAP1/HT-29 and lv-YAP1/Caco-2 cells and their control cells. **(B)** YAP1 protein expression in lv-YAP1/HT-29 and lv-YAP1/Caco-2 cells and their control cells; β-actin was used as control. (**C and D**) The proliferation rates of lv-YAP1/HT-29 (C) and lv-YAP1/Caco-2 cells (D) and their control cells from days 1 through 4 after seeding (^*^: *P<* 0.05, ^**^: *P<*0.01). **(E)** The colony formation assays indicated that more colonies were formed in lv-YAP1/HT-29 and lv-YAP1/Caco-2 cells compared to their respective control cells (^**^: *P<* 0.01). **(F)** Flow cytometry of in lv-YAP1/HT-29, lv-YAP1/Caco-2 cells and their control cells. **(G)** Tumorsphere formation in lv-YAP1/HT-29, lv-YAP1/Caco-2 cells and their control cells. **(H)** The cell numbers per tumorsphere in lv-YAP1/HT-29 and lv-YAP1/Caco-2 cells were significantly higher than their respective control cells (^**^: *P<* 0.01).

To further determine whether YAP1 could ensure the self-renewability of colon cancer progenitor cells, we established YAP1-enforced HT-29 (lv-YAP1/HT-29) and Caco-2 (lv-YAP1/Caco-2) cell lines with lentivirus particles using an LV5 (EF-1aF/GFP & Puro) vector with a YAP1 insert. YAP1 relative mRNA and protein levels in lv-YAP1/HT-29 and lv-YAP1/Caco-2 cells were significantly increased compared with their respective controls (Figures 4A and 4B). The proliferation rates of lv-YAP1/HT-29 and lv-YAP1/Caco-2 cells from days 3 through 4 after seeding were significantly increased than their respective control cells (Figures 4C and 4D). More colonies were formed in lv-YAP1/HT-29 and lv-YAP1/Caco-2 cells than their respective control cells (Figure 4E). In YAP1-enforced HT-29 or Caco-2 cells, 65.5% and 73.3% of the population were CD133^+^CD44^+^ when compared with 53.1% and 50.2% of their control cell population being CD133^+^CD44^+^ (Figure 4F). Larger tumorspheres were formed in lv-YAP1/HT-29 and lv-YAP1/Caco-2 cells compared with their respective control cells, and the cell numbers per tumorsphere in lv-YAP1/HT-29 and lv-YAP1/Caco-2 cells were significantly higher than their respective control cells (Figure 4G and 4H).

### YAP1-enforced expression in HT-29 and Caco-2 cells increased Ascl2, KLF5 and ‘stemness’-associated genes expression which were reversed by Ascl2 knockdown

**Figure 5:**
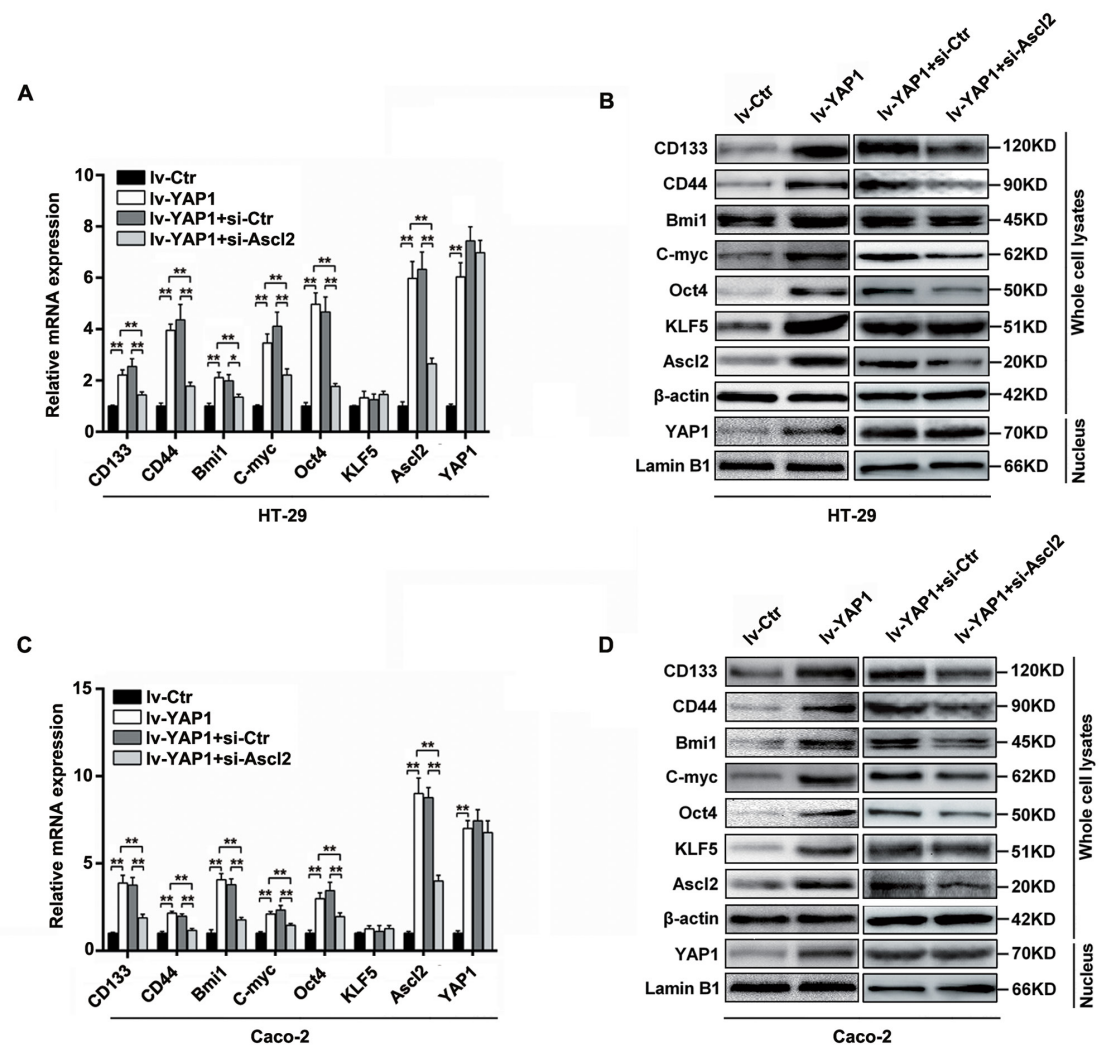
YAP1-enforced expression in HT-29 and Caco-2 cells increased Ascl2, KLF5 and ‘stemness’-associated genes expression, which were attenuated by Ascl2 knockdown (**A and B**) Relative Ascl2, KLF5 and ‘stemness’-associated genes expression levels in both mRNA (A) and protein (B) in YAP1 enforced expressed HT-29, and further Ascl2 interfered lv-YAP1/HT-29 cells. (**C and D**) Relative Ascl2, KLF5 and ‘stemness’-associated genes expression levels in both mRNA (C) and protein (D) in YAP1 enforced expressed Caco-2, and further Ascl2 interfered lv-YAP1/ Caco-2 cells. YAP1 nuclear accumulation was based on the blotting using the extracted nuclear proteins in relative cells. β-actin was used as a loading control, Lamin B1 was used as an internal control for the nuclear fraction.

To confirm whether the YAP1-enhanced self-renewability of colon cancer progenitor cells was related to a KLF5-dependent Ascl2 increase, the relative ‘stemness’-associated genes expression (mRNA) levels and protein levels in lv-YAP1/HT-29 and lv-YAP1/Caco-2 cells were determined, and they were found to be significantly increased compared to their respective control cells (Figure 5). The KLF5 mRNA levels were unaltered, but its protein levels were increased, and it is reported that KLF5 degradation could be prevented by increased YAP1 expression [39-40]. Ascl2 mRNA and protein expression levels were increased significantly in lv-YAP1/HT-29 and lv-YAP1/Caco-2 cells compared with their respective control cells (Figures 5). YAP1 nuclear translocation and accumulation were predominant in both lv-YAP1/HT-29 and lv-YAP1/Caco-2 cells (Figure 5C). The results indicated that YAP1 overexpression in HT-29 and Caco-2 cells increased Ascl2 and ‘stemness’-associated genes expression and KLF5 protein level.

To investigate whether Ascl2 mediated YAP1-induced ‘stemness’-associated genes expression, we performed Ascl2 interference in lv-YAP1/HT-29 and lv-YAP1/Caco-2 cells. The Ascl2-interfered lv-YAP1/HT-29 or lv-YAP1/Caco-2 cells exhibited a significant reversal in ‘stemness’-associated genes expression compared their control cells (Figure 5).

### YAP1 and KLF5 combined and bound to Ascl2 promoter

**Figure 6:**
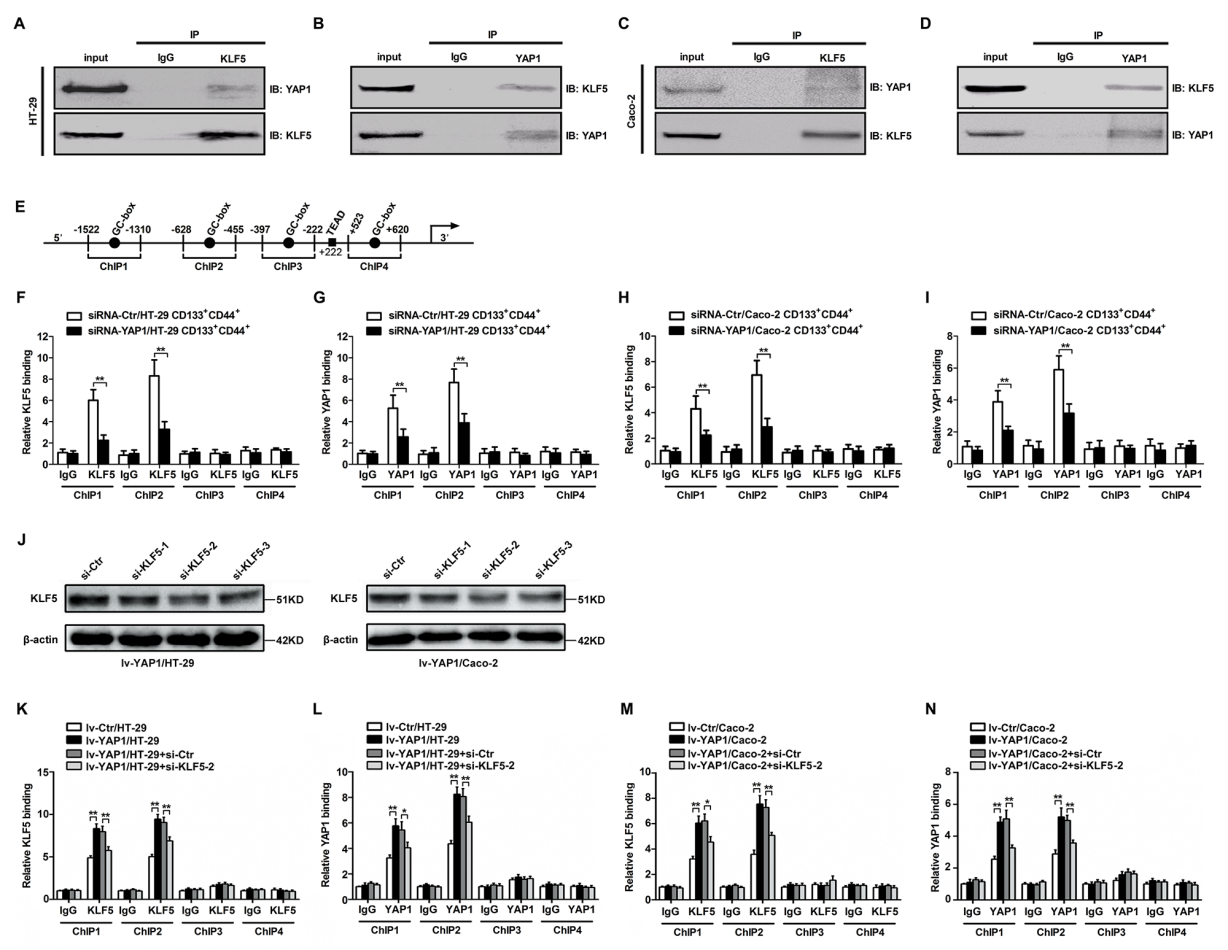
YAP1 and KLF5 combined and bound to the human Ascl2 promoter (**A-D**) Co-immunoprecipitation using anti-KLF5 (A and C) or anti-YAP1 (B and D) antibodies in the cell lysates from HT-29 (A and B) and Caco-2 (C and D) cells indicated that the immunoprecipitants of anti-KLF5 or anti-YAP1 antibodies in HT-29 and Caco-2 cells can be detected by both anti-KLF5 and anti-YAP1 antibodies. **(E)** A schematic representation of the human Ascl2 promoter. Four loci in the Ascl2 promoter that had a GC-box mutant (AAGTAG) (ChIPs 1-4) were tested and there was a potential TEAD (TEA domain family members (TEAD1-TEAD4)) binding sequence residing at +222 in the Ascl2 promoter. (**F and G**) Chromatin isolated from YAP1-interfered CD133^+^CD44^+^ HT-29 cell population and their control cells were subjected to immunoprecipitation using IgG and a rabbit polyclonal IgG against KLF5 (F) and IgG and a rabbit polyclonal IgG against YAP1 (G). The binding at the first two loci that had GC-boxes in YAP1-interfered CD133^+^CD44^+^ HT-29 cell population was significantly reduced compared with the control cells (^**^: *P<*0.01). (**H and I**) ChIP assays using YAP1-interfered CD133^+^CD44^+^ Caco-2 cell population and their control cells indicated that both KLF5 and YAP1 binding at the first two loci that had GC-boxes in YAP1-interfered CD133^+^CD44^+^ HT-29 cell population were significantly reduced compared with the control cells (^**^: *P<*0.01). **(J)** Western blots of KLF5 in lv-YAP1/HT-29 and lv-YAP1/Caco-2 cells that were transiently transfected with KLF5 siRNA1 (si-KLF5-1), siRNA2 (si-KLF5-2) or siRNA3 (si-KLF5-3); β-actin was used as a loading control. (**K-N**) ChIP assays using lv-YAP1/HT-29 and si-KLF5-2 transfected lv-YAP1/HT-29 cells (K and L), and lv-YAP1/Caco-2 and si-KLF5-2 transfected lv-YAP1/Caco-2 cells (M and N) cells and their control cells indicated that both KLF5 and YAP1 binding at the first two loci that had GC-boxes in lv-YAP1/HT-29 and lv-YAP1/Caco-2 cells was significantly increased compared with their respective control cells, KLF5 interference in lv-YAP1/HT-29 and lv-YAP1/Caco-2 cells led to the significant reduction of KLF5 or YAP1 binding to the human Ascl2 promoter (^*^: *P<*0.05, ^**^: *P<*0.01).

There were four loci in the Ascl2 promoter that had a GC-box (GGGCGG), which are potential binding sites for KLF5 [41]. YAP1 is a transcriptional co-activator and has been reported to bind with KLF5 in breast cells [40]. We performed the co-immunoprecipitation assay using anti-KLF5 or anti-YAP1 antibodies, the immunoprecipitants of anti-KLF5 or anti-YAP1 antibodies in HT-29 and Caco-2 cells can be detected by both anti-KLF5 and anti-YAP1 antibodies (Figures 6A-6D). Four loci within the Ascl2 promoter that had a GC-box (GGGCGG) were selected for chromatin immunoprecipitation (ChIP) assay. Chromatin isolated from YAP1-interfered CD133^+^CD44^+^ HT-29 or Caco-2 cell population and their control cells was subjected to immunoprecipitation using IgG and a rabbit polyclonal IgG against KLF5 and IgG and a rabbit polyclonal IgG against YAP1. Binding at the first two loci that contained a GC-box in YAP1-interfered CD133^+^CD44^+^ HT-29 or Caco-2 cell population was significantly reduced compared with their respective control cells (Figures 6F-6I). In order to confirm whether KLF5 depletion reduces YAP1 binding and Ascl2 luciferase reporter activity upon YAP1 overexpression, we firstly observed the interference efficiency of three different KLF5 siRNAs in Lv-YAP1/HT-29 or Lv-YAP1/Caco-2 cells and confirmed si-KLF5-2 was the most efficient sequence to inhibit KLF5 expression (Figure 6J). ChIP assays using lv-YAP1/HT-29, lv-YAP1/Caco-2 cells and their respective control cells indicated that both KLF5 and YAP1 binding at the first two loci that had a GC-box was significantly increased compared with control cells (Figures 6J-6M). ChIPs 3 and 4 in both YAP1-interfered CD133^+^CD44^+^ CRC cell population and YAP1-enforced expressed CRC cells gave negative results, which indicated that both KLF5 and YAP1 bound to the first two loci that had a GC-box, but did not bind the other two loci. KLF5 knockdown in Lv-YAP1/HT-29 or Lv-YAP1/Caco-2 cells reversed the increased YAP1 or KLF5 binding to Ascl2 promoter upon YAP1 overexpression (Figures 6K-6N).

### Transcriptional regulation of Ascl2 by YAP1

**Figure 7:**
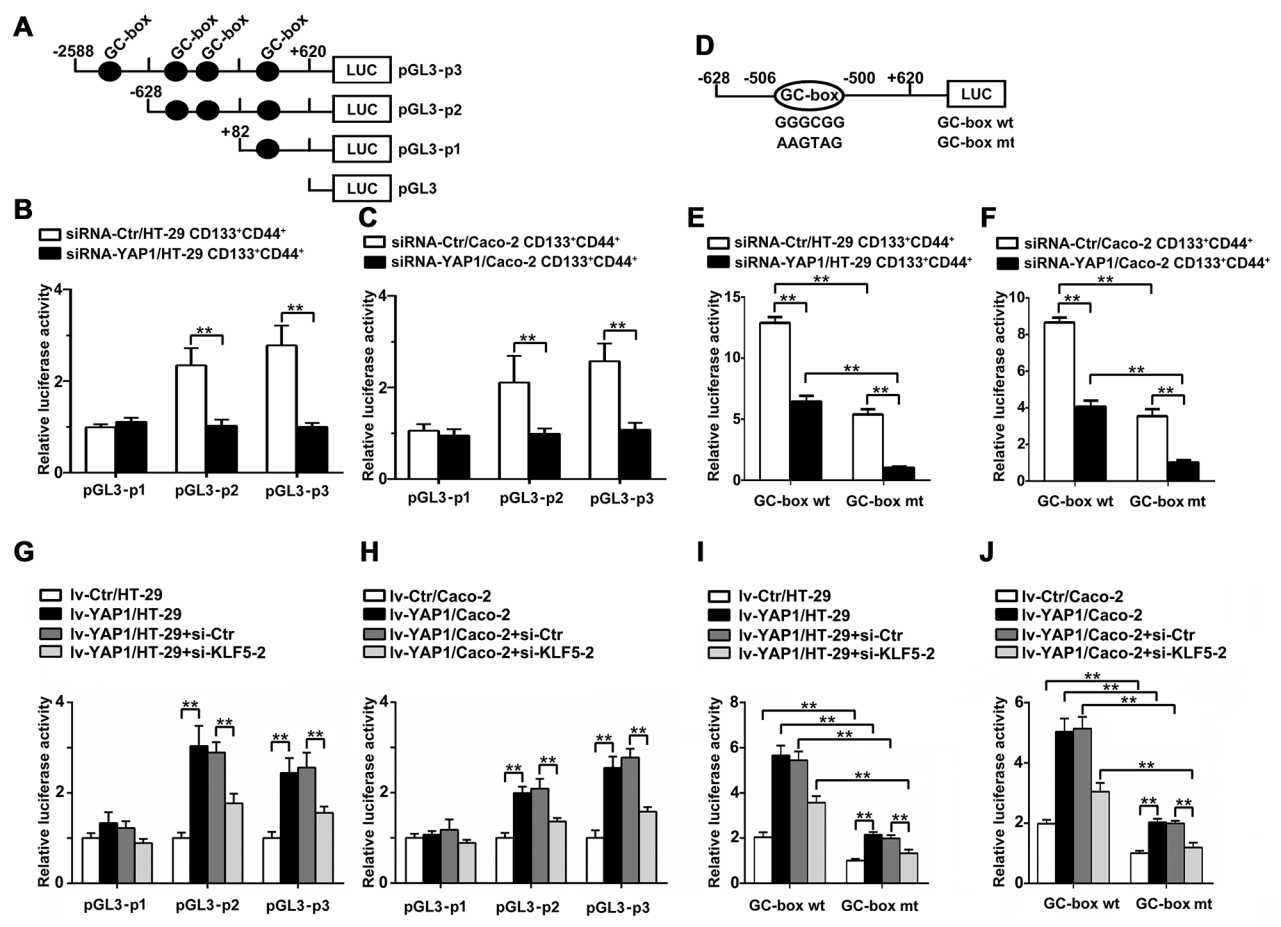
Transcriptional regulation of the Ascl2 gene in YAP1-interfered CD133^+^ CD44^+^ CRC cell population or YAP1-enforced expressed HT-29 or Caco-2 cells We performed luciferase assays using YAP1-interfered CD133^+^CD44^+^ CRC cell population to analyze the transcriptional regulation of the Ascl2 gene. **(A)** We produced a series of pGL3-Basic firefly luciferase reporters containing different human Ascl2 promoters encompassing bp -2588 to +620 (pGL3-p3), -628 to +620 (pGL3-p2), and -82 to +620 (pGL3-p1). The black dots represent GC-boxes. (**B and C**) A reduction of Ascl2 promoter activity was evident from the luciferase reporter assays in YAP1-interfered CD133^+^CD44^+^ HT-29 (B) and Caco-2 (C) cell population compared with their controls (n=3) (^**^: *P<*0.01). **(D)** A schematic representation of human Ascl2 promoter (-628 to +620) constructs containing the second potential KLF5 binding site, GC-box (GGGCGG) (GC-box wt), that was used in this study; the GC-box was mutated to GC-box mutant (AAGTAG) (GC-box mt). (**E and F**) YAP1-interfered CD133^+^CD44^+^ HT-29 (E) and Caco-2 (F) cell population and their control cells were transfected with the Ascl2 promoter-Luc construct (GC-box wt) or its mutant (GC-box mt). Reduced Ascl2 promoter activity was evident based on luciferase reporter assays (n=3) (^**^: *P<*0.01). (**G and H**) A significant increase in Ascl2 promoter activity was evident based on the luciferase reporter assays in lv-YAP1/HT-29 (G) and lv-YAP1/Caco-2 (H) cells compared with their controls (n=3), KLF5 interference in lv-YAP1/HT-29 (G) and lv-YAP1/Caco-2 (H) cells led to the significant reduction of Ascl2 promoter activity compared with their control cells (^**^: *P<*0.01). (**I and J**) The lv-YAP1/HT-29 (I) and lv-YAP1/Caco-2 (J) cells and their control cells were transfected with the Ascl2 promoter-Luc construct (GC-box wt) and its mutant (GC-box mt). GC-box mutation led to the significant reduction of Ascl2 promoter activities based on the luciferase reporter assays (n=3), KLF5 interference in lv-YAP1/HT-29 (I) and lv-YAP1/Caco-2 (J) cells led to further reduction of Ascl2 promoter activity compared with their control cells which were transfected with the Ascl2 promoter-Luc construct (GC-box wt) or its mutant (GC-box mt) (^**^: *P<*0.01).

We produced a series of pGL3-Basic firefly luciferase reporters containing different truncated versions of Ascl2 promoter (Figure 7A). A reduction in Ascl2 promoter activity was evident from the luciferase reporter assay involving Ascl2 promoter (-2588/+620) in YAP1-interfered CD133^+^CD44^+^ HT-29 and Caco-2 cells compared with their respective controls (Figures 7B and 7C). Then, we compared the abilities of the truncated promoters to drive Ascl2 expression in YAP1-interfered CD133^+^CD44^+^ HT-29 and Caco-2 cells, and significantly lower luciferase activity was observed in YAP1-interfered CD133^+^CD44^+^ HT-29 and Caco-2 cells when using the pGL3-Ascl2 promoter (-628/+620). However, no difference in luciferase activity using the pGL3-Ascl2 promoter (-82/+620) (Figures 7B and 7C) was observed. The results indicated that the first three GC-boxes might be responsible for the transcriptional regulation of the Ascl2 gene, and only the region containing the first two GC-boxes can be bound by KLF5 and YAP1 based on ChIP assays. Thus, the first two GC-boxes in the Ascl2 promoter region were used for the transcriptional regulation of Ascl2 by KLF5 and YAP1.

The inserted Ascl2 promoter in pGL3 vector (wild-type reporter, GC-box wt, pGL3-p2) contained a second potential KLF5 binding site (GGGCGG), and the second GC-box was mutated to GC-box mutant (AAGTAG) (GC-box mt) (Figure 7D). YAP1-interfered CD133^+^CD44^+^ HT-29 and Caco-2 cells and their control cells were transfected with Ascl2 promoter-Luc construct (GC-box wt) and its mutant (GC-box mt) and there were reduced Ascl2 promoter activities in YAP1-interfered CD133^+^CD44^+^ HT-29 and Caco-2 cells compared with their control cells, GC-box mt led to a significant reduction of Ascl2 promoter activities in both YAP1-interfered CD133^+^CD44^+^ HT-29 and Caco-2 cells and their control cells (Figures 7E and 7F).

A significant increase of Ascl2 promoter activity when using pGL3-p2 or pGL3-p3 was evident in lv-YAP1/HT-29 and lv-YAP1/Caco-2 cells compared with their control cells. KLF5 knockdown in Lv-YAP1/HT-29 and Lv-YAP1/Caco-2 cells reversed the Ascl2 promoter activities upon YAP1 overexpression (Figures 7G and 7H). GC-box mutation led to a significant reduction of Ascl2 promoter activities in Lv-YAP1/HT-29 and Lv-YAP1/Caco-2 cells, and KLF5 knockdown using si-KLF5-2 further reduced Ascl2 promoter activities (Figures 7I and 7J).

### Correlation between Ascl2 and YAP1 expression levels in CRC samples

**Figure 8:**
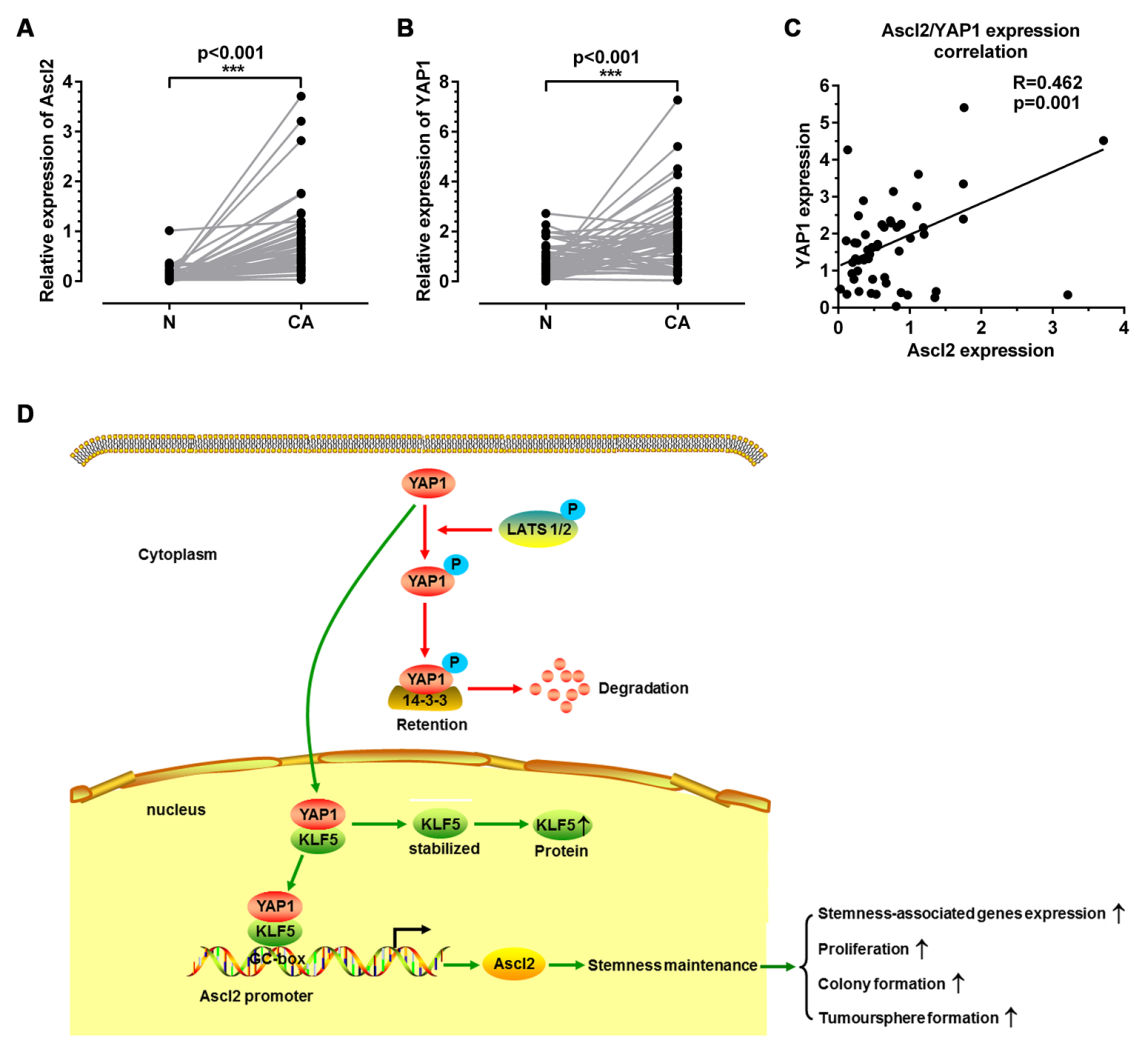
Correlation between Ascl2 and YAP1 mRNA levels in human colorectal carcinoma tissues and a working model of how YAP1 activated Ascl2 expression and ensured the self-renewability of colon cancer progenitor cells Quantitative real-time PCR was performed in CRC tissues (CA) and their peri-cancerous mucosa (N) to assess Ascl2 and YAP1 KLF5 mRNA levels. Ascl2 **(A)** and YAP1 **(B)** mRNA levels were higher in CRC samples when compared with those in the peri-cancerous mucosa. Ascl2 expression in CRC tissues was positively correlated with YAP1 expression (*P*=0.001) **(C)**. A working model detailing YAP1 activated Ascl2 and ensured the self-renewability of colon cancer progenitor cells. YAP1 was either modified by phosphorylation or not (Phosphorylated YAP1 was represented via the red route, whereas YAP1 without phosphorylation was represented via the green route) **(D)**.

To verify whether Ascl2 expression levels in human CRC samples were related to YAP1 expression levels, quantitative real-time PCR was used to quantify Ascl2 mRNA as well as YAP1 mRNA levels in 52 CRC samples and their normal mucosa. Ascl2 and YAP1 mRNA levels in cancerous tissues were significantly higher than those in normal mucosa (Figures 8A and 8B). The Ascl2 mRNA expression was correlated with YAP1 expression (Figure 8C) in human CRC samples.

## DISCUSSION

We demonstrated for the first time that the Hippo coactivator YAP1 was a molecular conveyor of self-renewal properties in CRC progenitor cells and Ascl2 can be activated by the Hippo signaling pathway in CRC progenitor cells, which ensures the self-renewability of CRC progenitor cells. YAP1 directly activated Ascl2 transcription in concert with its sequence-specific DNA binding partner KLF5.

The Hippo signaling pathway is gaining recognition as an important player in both organ size control and carcinogenesis because the disruption of the core kinase cascade initiated by Hippo and the phosphorylation of YAP/TAZ (Mst1/2, Sav1 and Lats1/2 and YAP1) can lead to carcinogenesis [42-44]. YAP1, an effector of the Hippo signaling pathway, has been reported as an oncogene in several tumor types [45-47]. TAZ (a transducer of the Hippo pathway) is required for breast CSCs to sustain self-renewal and tumor-initiation capacities [48]. The reciprocal regulation of tumor-initiating stem-like cells by TLR4, and TGF-β requires YAP1 and IGF2BP3 in hepatocellular carcinoma (HCC) [49]. Although YAP binding with β-catenin and TBX5 is thought as being central to the mechanism for YAP1-mediated regulation of Wnt/β-catenin signaling [34, 36], our observations in this study identified a novel mechanism that YAP1 could confer CRC progenitor cell properties via combination with KLF5 and bound to the KLF5 binding site (GC-box) in the Ascl2 promoter induced its transcriptional activation (Figure 8D).

Ascl2 plays a critical role in intestinal stem cells, CRC progenitor cells and human colorectal cancer [10-14] and is also overexpressed in gastric cancer and lung squamous cell carcinoma. It can accelerate cell growth, increase tumor resistance to 5-FU, and be regarded as an independent prognostic indicator [50, 51]. We have now provided evidence that YAP1-activated Ascl2 confered the acquisition of YAP1-induced CRC progenitor cell self-renewal properties. YAP1 and Ascl2 may facilitate transformation by the co-regulation of downstream targets or by the activation of distinct targets that synergize to promote the transformed phenotype.

The canonical Wnt/β-catenin signaling regulates many biological processes, including cell proliferation, cell fate decision, axis formation, and organ development during embryonic development and tissue homeostasis, it plays a critical roles as a driver of colon cancer [8, 10]. RNF43, ZNRF3, RSPO2 or RSPO3 alterations in breast, colorectal, gastric, pancreatic and other cancers activate the Wnt/β-catenin signaling. Gain-of-function mutations in the CTNNB1 gene, as well as loss-of-function mutations in the APC and AXIN2 genes, activate the canonical Wnt/β -catenin signaling cascade that regulates self-renewal, survival, proliferation and differentiation of tumor cells [52]. It is known that YAP1/TAZ can regulates Wnt/β-catenin signaling via binding with Dishevelled, β-catenin, or TBX5, or inducing the Wnt inhibitor DKK1 [34, 36, 52]. These reports suggest that the Hippo signaling pathway regulates Wnt/β-catenin signaling through multiple mechanisms, depending on biological context.

As a coactivator, YAP1 has been reported to regulate a series of genes, such as EGFR, ITGB2, CTGF, SOX9, ALPP, PIK3C2B, AREG, and BIRC5 [48, 53, 54]. The similarity of these studies is that YAP1-regulated transcription depends on transcription factor partners, such as TEAD, p73, ErbB4, and RUNX2 [55]. In this study, our initial observation is that YAP1 combined with KLF5 in HT-29 and Caco-2 cells, bound to the proximal Ascl2 promoter and induced its transcriptional activation. YAP1-enforced expression in HT-29 and Caco-2 cells did not increase KLF5 mRNA levels but significantly increased KLF5 protein levels, this result indicated that YAP1 did not transcriptionally induce KLF5 expression but possibly prevented KLF5 degradation, which is supported by a series of studies [39, 40]. KLF5 is an important transcription factor that is involved in regulating a larger number of genes, and KLF5 potentially functions as an oncogene [56]. Multicellular tumor spheroid (MCTS) formation in HT-29 colon carcinoma cells required high KLF5 expression, and tumor formation can be inhibited by KLF5 knockdown [57]. We analysed the Ascl2 promoter (-2588∼+620) and found a potential TEAD (TEA domain family members (TEAD1-TEAD4)) binding sequence residing at +222 of Ascl2 promoter (Figure 6E). This potential TEAD sequence is not involved with the activation of Ascl2 gene upon YAP1 overexpression, because the Ascl2 luciferase reporter activity (Figure 7A-7C) was unaltered although YAP1 overexpression,

The accordant increase of YAP1 and Ascl2 in human colon cancer samples was consistent with the observations *in vitro*. This indirect *in vivo* evidence, together with the *in vitro* results, strengthens our hypothesis for a role of YAP1 in the regulation of Ascl2.

Taken together, we suggest that YAP1/KLF5-activated Ascl2 expression in colon cancer progenitor cells confers their self-renewability. This novel mechanism provides a possible target for colon cancer progenitor cells.

## MATERIALS AND METHODS

### Cell culture

The HT-29 and Caco-2 human colonic adenocarcinoma cell lines were obtained from the American Type Culture Collection (ATCC) and routinely cultured in McCoy’s 5A medium (Sigma, USA) supplemented with 10% fetal bovine serum (FBS; HyClone, USA) at 37°C in a 5% CO_2_ humidified incubator.

### Flow cytometry cell sorting and flow cytometry analysis

The single-cell suspensions of HT-29 and Caco-2 cells were incubated with APC-conjugated anti-human CD133 antibody (Miltenyi Biotec) and FITC-conjugated anti-human CD44 antibody (Miltenyi Biotec) in PBS for 10 min at 4°C. The APC-conjugated mouse IgG2b isotype control antibody (Miltenyi Biotec) and the FITC-conjugated mouse IgG1 isotype control antibody (Miltenyi Biotec) served as negative controls. The CD133^+^/CD44^+^ and CD133^-^/CD44^-^ cell population was sorted using a fluorescence-activated cell sorter (BD Aria, USA).

### Cell proliferation

Isolated cells were seeded at 10 000 cells/well in 24-well plates. Then, 20 μl of CCK-8 solution (Beyotime Biotechnology, China) was added to each well and cultured at 37°C for 4 h. On the 1st, 2nd, 3rd and 4th days, the absorbance was measured using a microplate reader (Thermo, USA) at 450 nm. The experiments were repeated in triplicate.

### Colony formation assay

An equal number of viable cells were seeded at a density of 1 000 cells/well in 6-well plates. After incubating for 14 days at 37°C, the colonies that adhered to the bottoms of the plates were counted using an inverted microscope (Olympus, Japan).

### Tumorsphere formation

To evaluate the tumorsphere-formation potential, when single cells reached a density of 1 000 cells, they were plated in 48-well ultra-low attachment plates (Corning Inc, USA) with DMEM/F12 (HyClone, USA) serum-free media supplemented with B-27 (Gibco, USA), epidermal growth factor (EGF, Peprotech, USA) and basic fibroblast growth factor (bFGF, Peprotech, USA). Every 3 days, fresh medium was added. The experiment was terminated after 7 days, and spheres were quantified using an inverted microscope (Olympus, Japan).

### Real-time PCR

To quantitatively determine the expression levels of each gene, real-time PCR was performed using first-strand cDNA, forward and reverse primers and the SYBR premix Ex TaqTM Green II (TaKaRa, Japan). The primer sequences are summarized in Table 1, and β-actin was used as an endogenous control. Expression levels were analyzed using the comparative Ct method (2^-∆∆Ct^). The real-time PCR reactions were independently performed in triplicate.

**Table 1:** Sequences of the oligonucleotides used for qPCR and chromatin immunoprecipitation (ChIP)

qPCR	5’→ 3’
Ascl2	F: 5’-CGTGAAGCTGGTGAACTTGG-3’ R: 5’-GGATGTACTCCACGGCTGAG-3’
CD133	F: 5’-TTCTTGACCGACTGAGACCCA-3’ R: 5’-TCATGTTCTCCAACGCCTCTT-3’
CD44	F: 5’-CTGCCGCTTTGCAGGTGTA-3’ R: 5’-CATTGTGGGCAAGGTGCTATT-3’
Bmi1	F: 5’-CGTGTATTGTTCGTTACCTGGA-3’ R: 5’-TTCAGTAGTGGTCTGGTCTTGT-3’
C-myc	F: 5’-GGCTCCTGGCAAAAGGTCA-3’ R: 5’-CTGCGTAGTTGTGCTGATGT-3’
Oct4	F: 5’-CTGGAGAAGGAGAAGCTGGA-3’ R: 5’-CAAATTGCTCGAGTTCTTTCTG-3’
KLF5	F: 5’-AAGGAGTAACCCCGATTTGG-3’ R: 5’-TGGCTTTTCACCAGTGTGAG-3’
YAP1	F: 5’-TAGCCCTGCGTAGCCAGTTA-3’ R: 5’-TCATGCTTAGTCCACTGTCTGT-3’
MST1	F: 5’-AGTGCCAAAGGAGTGTCAATAC-3’ R: 5’-GGATTCCTGGCGTTTCAGTTTC-3’
β-actin	F: 5’-TTGCCGACAGGATGCAGAA-3’ R: 5’-GCCGATCCACACGGAGTACT-3’
**ChIP**	**5’→ 3’**
ChIP1	F: 5’-TCAGACCAGGGACAGGGAG-3’ R: 5’-GGCCGGAAGAGTAGCACC-3’
ChIP2	F: 5’-CTGGCACAAGGCGGTCGG-3’ R: 5’-TGAGGCGGGGTGGGAGGTT-3’
ChIP3	F: 5’-CCCAGTAGATTAACGCACAG-3’ R: 5’-GCCCCATTGAGGAAGC-3’
ChIP4	F: 5’-GCCACAGTTTTCCCCGTCGCCTCC-3’ R: 5’-GCATCCACCCGCCCGCTCCA-3’
GAPDH (ChIP for positive control)	F: 5’-TACTAGCGGTTTTACGGGCG-3’ R: 5’-TCGAACAGGAGGAGCAGAGAGCGA-3’

### Western blotting

The cytoplasmic proteins and nuclear proteins were extracted using NE-PER nuclear and cytoplasmic extraction reagents (Thermo, USA). Separated protein bands were transferred onto PVDF membrane, and the membrane were probed overnight at 4°C with a primary antibody (Table 2). Protein bands were visualized using the chemiluminescent Pierce ECL kit (Thermo Scientific), and quantification of the Western blots was performed using ChemiDox™ XRS with Image Lab™ software. Detailed Western blotting procedures have been described previously [58].

**Table 2:** The primary antibodies used in the experiment

Primary antibody	Company	Dilution
Mouse monoclonal IgG to Ascl2	Millipore(MAB4418)	WB: 1:500
Rabbit polyclonal IgG to CD133	Proteintech(18470-1-AP)	WB: 1:500
Rabbit polyclonal IgG to CD44	Proteintech(15675-1-AP)	WB: 1:200
Rabbit polyclonal IgG to Bmi1	Proteintech (10832-1-AP)	WB: 1:500
Rabbit polyclonal IgG to C-myc	Proteintech(10828-1-AP)	WB: 1:500
Rabbit polyclonal IgG to Oct4	Proteintech(11263-1-AP)	WB: 1:500
Rabbit polyclonal IgG to KLF5	Proteintech(21017-1-AP)	WB: 1:500
Rabbit polyclonal IgG to KLF5	Santa Cruz Biotechnology(sc-22797)	IP: 1:50; ChIP
Rabbit polyclonal IgG to YAP1	Proteintech(13584-1-AP)	WB: 1:500
Rabbit monoclonal IgG to YAP1	Cell Signaling(^#^14074)	IP: 1:50; ChIP
Rabbit polyclonal IgG to MST1	Cell Signaling(^#^3682)	WB: 1:1000
Rabbit monoclonal IgG to Phospho-YAP(Ser127)	Cell Signaling(^#^13008)	WB: 1:1000
Mouse monoclonal IgG to β-actin	Proteintech(66009-1-Ig)	WB: 1:4000
Rabbit polyclonal IgG to Lamin B1	Proteintech(12987-1-AP)	WB: 1:5000

### siRNA transfection

The CD133^+^/CD44^+^ and CD133^-^/CD44^-^ cell population from HT-29 or Caco-2 cells was seeded in 6-well plates and transfected with YAP1 siRNAs or the scrambled control, and the lv-YAP1/HT-29 and lv-YAP1/ Caco-2 cells were transfected with KLF5 siRNAs or Ascl2 siRNA and their scrambled controls using the FuGENE HD transfection reagent (Promega, USA). The siRNA sequences were designed by GenePharma Co. Ltd. (Shanghai, China). The sequences of the three YAP1 siRNAs were as follows: siRNA-YAP1-1, Forward: 5’-GGUGAUACUAUCAACCAAATT-3’, Reverse: 5’-UUUGGUUGAUAGUAUCACCTT-3’; siRNA-YAP1-2, Forward: 5’-CUGCCACCAAGCUAGAUAATT-3’, Reverse: 5’-UUAUCUAGCUUGGUGGCAGTT-3’; siRNA-YAP1-3, Forward: 5’-GACGACCAAUAGCUCAGAUTT-3’, Reverse: 5’-AUCUGAGCUAUUGGUCGUCTT-3’. The sequences of the three KLF5 siRNAs were as follows: si-KLF5-1, Forward: 5’-GCAGACUGCAGUGAAACAATT-3’, Reverse: 5’- UUGUUUCACUGCAGUCUGCTT-3’, si-KLF5-2, Forward: 5’- GGCAAUUCACAAUCCAAAUTT-3’, Reverse: 5’-AUUUGGAUUGUGAAUUGCCTT-3’, si-KLF5-3, Forward: 5’-GCAUCCACUACUGCGAUUATT-3’, Reverse: 5’-UAAUCGCAGUAGUGGAUGCTT-3’. Ascl2 siRNA was described in previously [23]. The cells were harvested 48 h after transfection and YAP1 or KLF5 expression was examined.

### YAP1 overexpression

Lentivirus particles expressing YAP1 were produced by GenePharma Co. Ltd. China. HT-29, and Caco-2 cells were transfected with the lentivirus particles using an LV5 vector with a YAP1 insert. Stably transfected cells with GFP were sorted using flow cytometry (BD FACS Aria II; BD Biosciences, Franklin Lakes, NJ, USA) and isolated using puromycin selection (Solarbio, Beijing, China).

### Co-immunoprecipitation

Cell lysates were immunoprecipitated with anti-KLF5 (Santa Cruz, SC22797) or anti-YAP1 (Cell Signaling, 14074) polyclonal antibodies according to the instructions of the BeaverBeads™ Protein A/G Immunoprecipitation Kit (22202-20), separated by SDS/PAGE and analyzed by Western blotting.

### ChIP assays

ChIP assays were performed using a ChIP assay kit (Millipore, 17408) according to the manufacturer’s instructions. Soluble chromatin was prepared from siRNA-YAP1-1/HT-29 CD133^+^/CD44^+^ or siRNA-YAP1-1/Caco-2 CD133^+^/CD44^+^ cell population and their control cells as well as lv-YAP1/HT-29 or lv-YAP1/ Caco-2 and their control cells. The chromatin was immunoprecipitated with anti-KLF5 (Santa Cruz, SC22797, USA) or anti-YAP1 (Cell Signaling, 14074, USA). The precipitated DNA was subjected to real-time PCR using specific primers. The primer sequences and the lengths of the amplified PCR products are shown in Table 3.

**Table 3:** The primer sequences used in the each Ascl2 promoter fragment

pGL3-Ascl2-promotor	5’→ 3’	Length of product
-2588/+620	F: 5’-ggggtacc TCCGGAGATCTTACCA-3’ R: 5’-cggctagc CGCGCCTGCATCCAC-3’	3208 bp
-628/+620	F:5’-ggggtacc GACCGTCCTGGCACAAG-3’ R: 5’-cggctagc CGCGCCTGCATCCAC-3’	1248 bp
-82/+620	F: 5’-ggggtacc GATCTTGCGCGCCTC-3’ R: 5’-cggctagc CGCGCCTGCATCCAC-3’	702 bp

The underlined parts in the primers are KpnI and NheI adaptors.

### Transfection and luciferase assays

The Ascl2 5′-flanking sequence (-2588/+620 bp region), amplified using PCR from intestinal tissue DNA, was inserted into the luciferase reporter vector pGL3-Basic (Promega, Madison, WI, USA). The primer pairs used to produce each promoter fragment are listed in Table 3. All transfections were performed using the FuGENE HD transfection reagent (Promega, USA). Luciferase activity was measured 48 h post-transfection using the Dual-Luciferase Reporter Assay System (Promega) as described in our previous report [59]. The pGL3-Basic vector containing the Ascl2 promoter (-628/+620) and one GC-Box site (GGGCGG) served as the wild-type construct used for the generation of the Ascl2-Luc mutant, which harbors a mutation in the GC-BOX site (AAGTAG) created by PCR-based site-directed mutagenesis. The transfection of the luciferase reporters containing the various Ascl2 promoters and the luciferase assays were performed as described in our previous report [17, 59].

### Tissue samples

A total of 52 patients with colorectal cancer who were scheduled for colonoscopy or for surgical resection were enrolled in this study. Cancerous samples and their peri-cancerous mucosa were collected by biopsy or from the resection samples. The fresh samples were immediately stored in liquid nitrogen for further quantitation of Ascl2, YAP1 and KLF5 mRNA levels using quantitative real-time PCR analysis. This study was approved by the local clinical research ethics committee. All the subjects provided informed consent prior to their colonoscopy or resection surgery.

### Statistical analysis

GraphPad Prism 6.0 software was used for all statistical analyses. The differences were deemed significant when *P<*0.05 and very significant when *P<*0.01.
